# Development of a micronucleus test using the EpiAirway™ organotypic human airway model

**DOI:** 10.1186/s41021-023-00269-2

**Published:** 2023-04-12

**Authors:** Satoru Munakata, Taku Watanabe, Tomohiro Takahashi, Shiori Kimuro, Kanae Ishimori, Tsuneo Hashizume

**Affiliations:** Scientific Product Assessment Center, Japan Tobacco Inc, 6-2, Umegaoka, Aoba-Ku, Yokohama, Kanagawa 227-8512 Japan

**Keywords:** Micronucleus test, Organotypic human airway model, EpiAirway™, EGF, Clastogens, Aneugen

## Abstract

**Background:**

The use of organotypic human tissue models in genotoxicity has increased as an alternative to animal testing. Genotoxicity is generally examined using a battery of in vitro assays such as Ames and micronucleus (MN) tests that cover gene mutations and structural and numerical chromosome aberrations. At the 7th International Workshop on Genotoxicity Testing, working group members agreed that the skin models have reached an advanced stage of maturity, while further efforts in liver and airway models are needed [Pfuhler et al., Mutat. Res. 850–851 (2020) 503135]. Organotypic human airway model is composed of fully differentiated and functional respiratory epithelium. However, because cell proliferation in organotypic airway models is thought to be less active, assessing their MN-inducing potential is an issue, even in the cytokinesis-blocking approach using cytochalasin B (CB) [Wang et al., Environ. Mol. Mutagen. 62 (2021) 306–318]. Here, we developed a MN test using EpiAirway™ in which epidermal growth factor (EGF) was included as a stimulant of cell division.

**Results:**

By incubating EpiAirway™ tissue with medium containing various concentrations of CB, we found that the percentage of binucleated cells (%BNCs) almost plateaued at 3 μg/mL CB for 72 h incubation. Additionally, we confirmed that EGF stimulation with CB incubation produced an additional increase in %BNCs with a peak at 5 ng/mL EGF. Transepithelial electrical resistance measurement and tissue histology revealed that CB incubation caused the reduced barrier integrity and cyst formation in EpiAirway™. Adenylate kinase assay confirmed that the cytotoxicity increased with each day of culture in the CB incubation period with EGF stimulation. These results indicated that chemical treatment should be conducted prior to CB incubation. Under these experimental conditions, it was confirmed that the frequency of micronucleated cells was dose-dependently increased by apical applications of two clastogens, mitomycin C and methyl methanesulfonate, and an aneugen, colchicine, at the subcytotoxic concentrations assessed in %BNCs.

**Conclusions:**

Well-studied genotoxicants demonstrated capability in an organotypic human airway model as a MN test system. For further utilization, investigations of aerosol exposure, repeating exposure protocol, and metabolic activation are required.

## Introduction

Over the last decade, the use of organotypic human tissue models in toxicology has increased as an alternative to animal testing [1, 2]. In vitro assays and animal models are recognized as being relatively effective in the detection of acute and severe toxicities; however, some studies demonstrated that animal experiments are poorly reproducible, and the significant physiological differences between humans and other species have raised concerns about the reliability of data derived from animal experiments [3–5]. Three-dimensional tissue models better mimic the structure and function of tissue compared with standard two-dimensional cell culture systems by supporting cell–cell interactions and signaling pathways, which makes organotypic skin and liver models more predictable in detecting the genotoxicity induced by various chemicals [6–9]. When organotypic human tissue models are validated, these can be used to evaluate the genotoxic effect of chemicals in relevant physiological circumstances in a cost-effective manner without the need to conduct animal experiments.

Genotoxicity assessment is a core requirement in regulatory toxicology, and it is a critical component of the risk assessment of all types of substances ranging from pharmaceuticals, industrial chemicals, and pesticides, to food additives [10]. Genotoxic potential is generally examined by a battery of in vitro assays covering the endpoints of gene mutations and structural and numerical chromosome aberrations, such as the Ames assay and the in vitro micronucleus test, respectively [11, 12]. At the 7th International Workshop on Genotoxicity Testing meeting in Tokyo, November 2017, a “Use of 3D Tissues in Genotoxicity Testing” working group (WG) discussed how 3D tissue models may be utilized in genotoxicity testing, and the WG considered that it was important that the full endpoints of genotoxic damage (i.e., mutagenicity, clastogenicity, and aneugenicity) can be detected in 3D human skin, liver, and airway models. The WG recognized that skin models have reached an advanced state of validation, while further efforts in liver and airway model-based assays are needed to cover the three main endpoints of genotoxicity [7].

Organotypic human airway models comprise fully differentiated and functional human respiratory epithelium including beating ciliated cells, mucus-producing goblet cells, and progenitor basal cells [13, 14]. Culturing organotypic airway models at an air–liquid interface (ALI) enables relevant exposure to air, i.e., inhalation. In human ALI airway models, several disease-relevant physiological and molecular tissue responses have been confirmed [15, 16]. However, cell proliferation in organotypic airway models is not thought to be active, creating an issue in assessing the genotoxic potential because fixed DNA damage occurs throughout DNA replication. Recently, Wang et al. reported detection of DNA damage by CometChip assay as well as mutagenicity by Duplex Sequencing, an error-corrected next-generation sequencing method, in organotypic human airway models treated with a well-studied genotoxicant, ethyl methanesulfonate [17]. In their study, a 28-day exposure schedule was employed to accumulate the fixed DNA damage (i.e., gene mutation) induced by ethyl methanesulfonate in an organotypic airway model. They chose a repeating exposure regimen because a previous study reported that only approximately 5% of the cells were stained with anti-Ki67 antibody, a marker of cell proliferation, in a commercially available organotypic airway model, MucilAir™ from Epithelix [18]. This suggests that the rate of cell division was too low to obtain a sufficient number of binucleated cells (BNCs), even when incubated with cytochalasin B (CB) [7].

According to the Organization for Economic Co-operation and Development (OECD) Test Guideline 487 [19], primary human peripheral blood lymphocytes require mitogenic stimulation by phytohemagglutinin to induce cell division prior to exposure to a test chemical in the micronucleus (MN) test. Primary rat hepatocytes [20] and human hepatoma HepaRG cells [21–23] also require mitogenic stimulation by epidermal growth factor (EGF) to undergo cell division for proper assessment of the potential to induce MN. Because it was reported that EGF induced cell proliferation and multilayered epithelium formation in primary normal human tracheobronchial epithelial cells cultured at ALI [24], we employed EGF stimulation to induce cell division for the assessment of the potential to induce MN by a genotoxicant in organotypic human airway models.

In the present study, we developed a MN test using the organotypic human airway model EpiAirway™ as a respiratory tissue system in which the tissues were treated with a genotoxicant, followed by incubating with medium supplemented with CB to accumulate BNCs that have undergone cell division and EGF to stimulate cell division. First, we optimized the conditions for CB incubation and then adjusted the EGF stimulation to capture a sufficient number of BNCs. After examining the effect of CB incubation and EGF stimulation on the physiological and structural characteristics as well as cytotoxicity of EpiAirway™ tissue, we investigated the capability of our organotypic human airway model through MN assessment using well-studied clastogens, mitomycin C (MMC) and methyl methanesulfonate (MMS), and a well-studied aneugen, colchicine (COL).

## Materials and methods

### Test chemicals and reagents

MMS, COL, CB, ethylenediaminetetraacetic acid (EDTA) solution (approximately 0.5 M), trypsin (0.25%)-EDTA (0.02%) in Hanks’ balanced salt solution, potassium chloride solution (0.075 M), acetic acid, and acridine orange were obtained from Sigma–Aldrich (St. Louis, MO, USA). MMC, dimethyl sulfoxide, methanol, distilled water, and 4% paraformaldehyde phosphate buffer solution were obtained from FUJIFILM Wako Pure Chemical (Osaka, Japan). Dulbecco’s phosphate buffered saline (DPBS), Dulbecco's modified Eagle medium (DMEM), and fetal bovine serum (FBS) were obtained from Thermo Fisher Scientific (Waltham, MA, USA). Recombinant human EGF was obtained from PeproTech (Cranbury, NJ, USA). Transepithelial electrical resistance (TEER) buffer was obtained from MatTek (Ashland, MA, USA). Stock solutions of CB were prepared in dimethyl sulfoxide and stored at − 30 °C. EGF, MMC, MMS, and COL were dissolved in distilled water and stored at − 30 °C before use.

### Cell culture

The EpiAirway™ tissue model (AIR-100) was obtained from MatTek (Ashland, MA, USA). It was created in situ by differentiating normal human bronchial epithelial cells on a semi-permeable membrane support (MatTek PermaCell Cell Culture Inserts, part No. CCI24-PTFE-0.4, 0.6 cm^2^) at an ALI. Tissues were cultured in EpiAirway™ Maintenance Medium (AIR-100-MM; MatTek) according to the manufacturer’s protocol in a humidified 37 °C incubator with 5% CO_2_. For short-term (< 2 day) culture, EpiAirway™ tissues were maintained at the ALI in 6-well plates, with 1 mL medium in the basal compartment. For longer-term culture according to the manufacturer’s recommendations, tissues were maintained at the ALI in 12-well plates with hang-top lids (HNG-TOP-12; MatTek), with 5 mL culture medium in the basal compartment.

### Treatment schedule

**Fig. 1:**
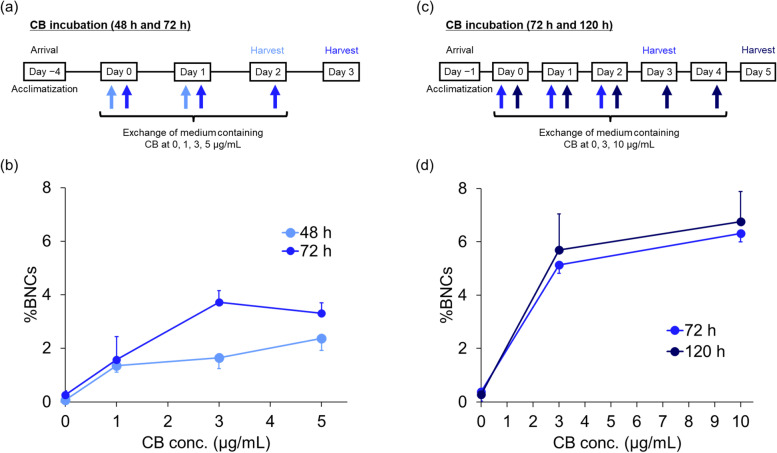
Optimization of incubation with cytochalasin B in the EpiAirway™ model. **a** Schematic of the incubation conditions with cytochalasin B (CB) for 48 or 72 h. **b** Percentage of binucleated cells (%BNCs) obtained after incubation with at 0, 1, 3, and 5 μg/mL for 48 h (in light blue) or 72 h (in blue). **c** Schematic of the incubation conditions with CB for 72 or 120 h. **d** %BNCs obtained after incubation with CB at 0, 3, and 10 μg/mL for 72 h (in blue) or 120 h (in deep blue). Values are means ± standard deviation of triplicate inserts

To optimize the incubation conditions with CB, EpiAirway™ tissue inserts were incubated with the culture medium, which contained several concentrations of CB for two incubation periods. As shown in Fig. 1a and c, on Day 0, the tissues on the insert were placed in 6-well culture plates and incubated with 1 mL/well of a culture medium containing a specific concentration of CB. For 48-h incubation, the basolateral medium was replaced once on Day 1 with medium containing CB, and cells were then harvested from the inserts on Day 2 (Fig. 1a). Similarly, for 72-h incubation, the basolateral medium was replaced twice on Days 1 and 2 with medium containing CB, and cells were then harvested from the inserts on Day 3 (Fig. 1a). Furthermore, the medium was also exchanged three times (from Days 0 to 2) or five times (from Days 0 to 4) at 24-h intervals for 72-h or 120-h incubation, respectively (Fig. 1c).

**Fig. 2:**
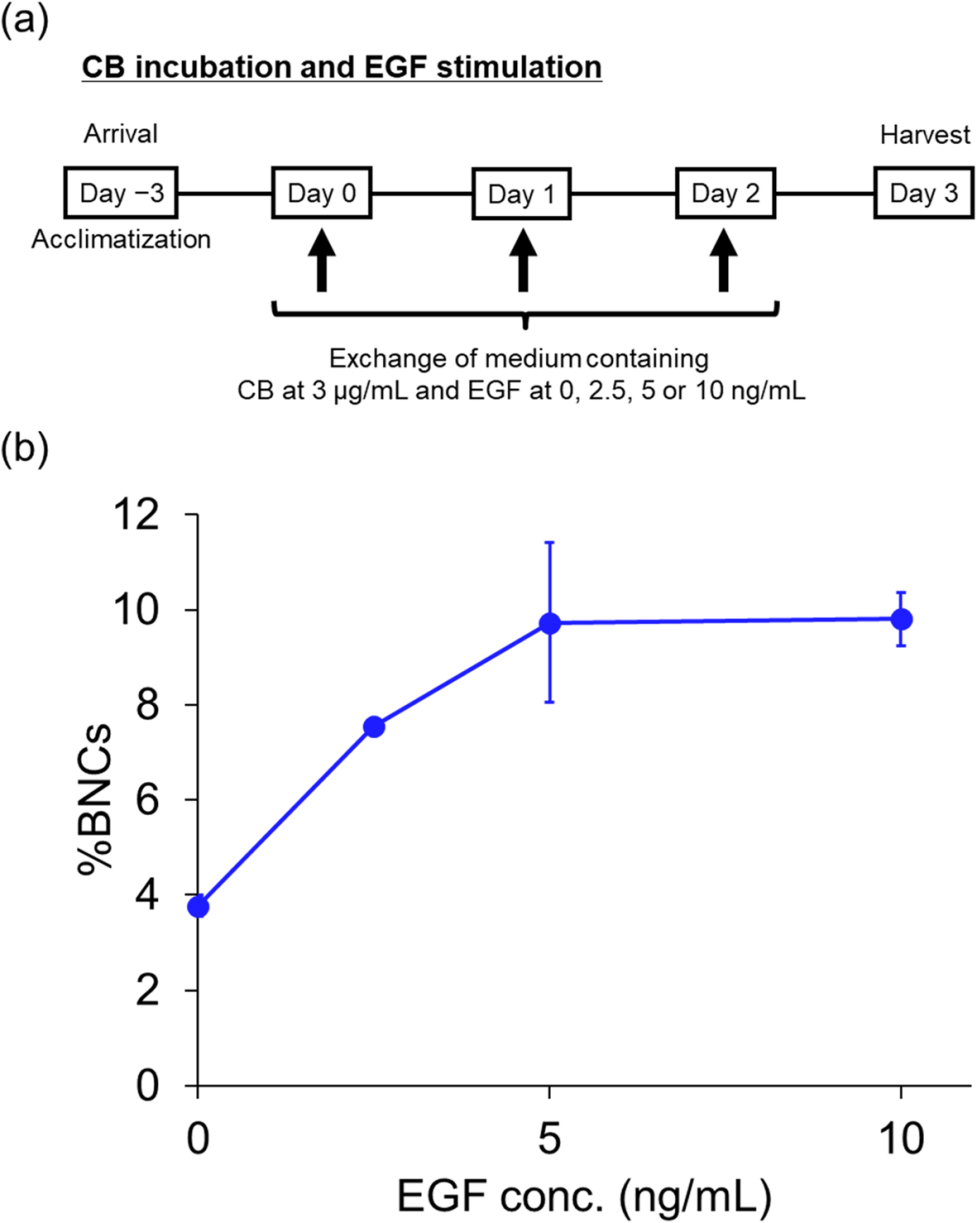
Effect of epidermal growth factor stimulation on the percentage of binucleated cells in EpiAirway™ model. **a** Schematic of epidermal growth factor (EGF) stimulation conditions for 72 h with cytochalasin B (CB) incubation. **b** Percentage of binucleated cells (%BNCs) obtained after stimulation with EGF at 0, 2.5, 5, and 10 ng/mL for 72 h with CB incubation. Values are means ± standard deviation of triplicate inserts, except for the 2.5 ng/mL EGF with CB incubation condition, for which values were derived from duplicate inserts

For CB incubation with EGF stimulation (Fig. 2a), the tissues on the insert were placed in 6-well culture plates and incubated with 1 mL/well of a culture medium containing a specific EGF with 3 μg/mL CB in the basolateral compartment of the well on Day 0. In this treatment schedule, the basolateral medium was replaced twice on Days 1 and 2. Cells were harvested from the inserts on Day 3.

**Fig. 3:**
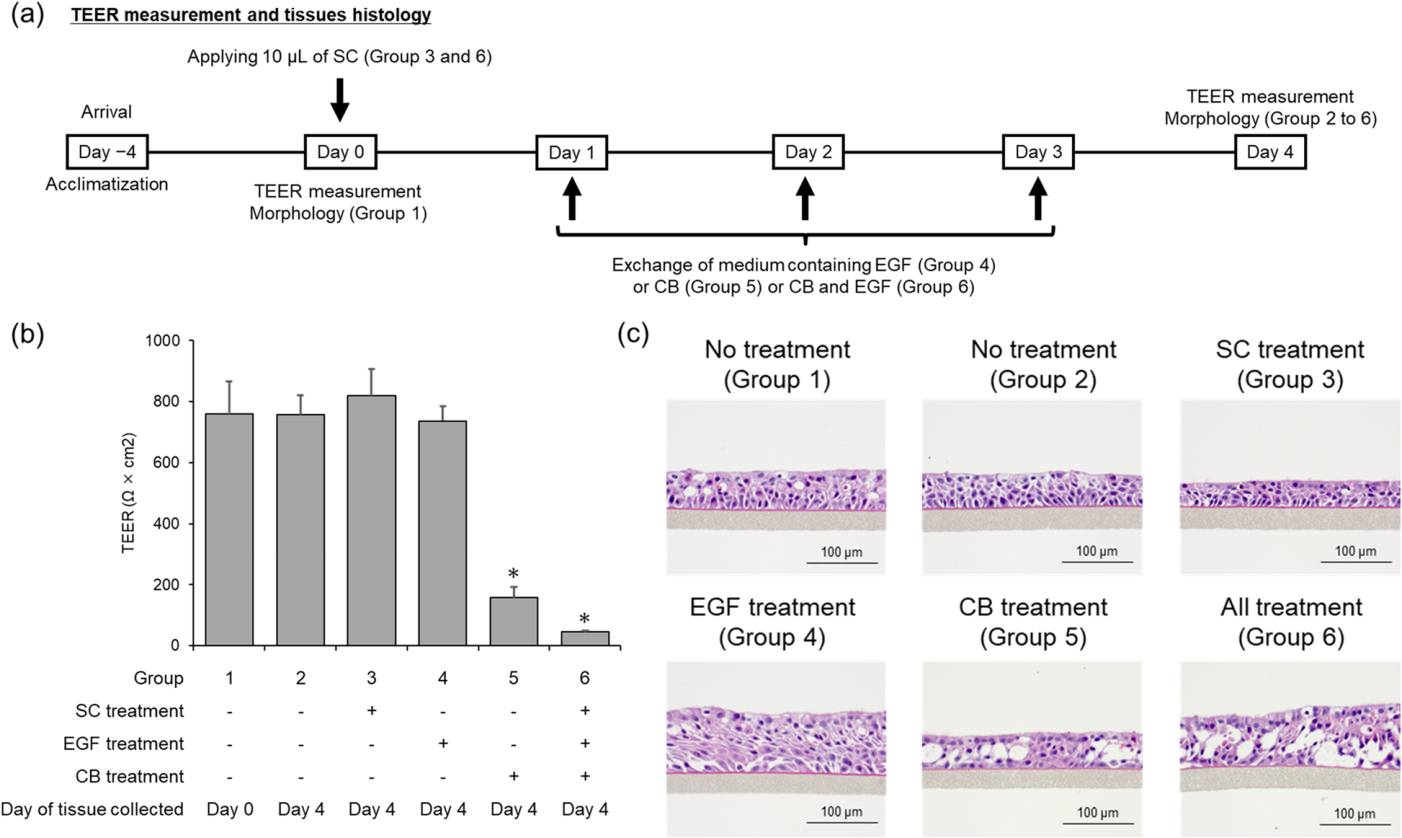
Changes in transepithelial electrical resistance and tissue morphology induced in various experimental conditions. **a** Schematic of transepithelial electrical resistance (TEER) measurement and histology of EpiAirway™ tissue under various experimental conditions. Group 1, immediately after a 3-day acclimatization period; Group 2, no test chemical treatment, cytochalasin B (CB) incubation, or epidermal growth factor (EGF) simulation throughout the experimental period; Group 3, solvent treatment; Group 4, no chemical treatment followed by EGF stimulation only; Group 5, no chemical treatment followed by CB incubation only; and Group 6, solvent treatment followed by CB incubation and EGF stimulation. **b** Tissue integrity assay. Values are means ± standard deviation of triplicate inserts. *Significantly different from the ‘Group 2’ (*p* < 0.05, Welch’s *t*-test). **c** Representative images of tissue sections stained with hematoxylin and eosin from samples collected on Day 0 and Day 4

As shown in Fig. 3a, to investigate the effect of various treatments (i.e., CB incubation, EGF stimulation, and test chemical treatment from the apical side of the tissue) on barrier function and morphology, TEER measurement and tissue histology were performed (six groups in total). The details of each group were as follows: Group 1, TEER measurement and tissue histology were performed immediately after a 3-day acclimatization period on Day 0; Group 2, tissues were incubated with the normal culture medium without test chemical, CB, and EGF throughout the experimental period, and TEER measurement and tissue histology were performed on Day 4; Group 3, 10 μL solvent control was applied with a micropipette directly to the apical surface of the tissue on Day 0; Group 4, tissues were treated with EGF only from Day 1 to Day 4; Group 5, tissues were treated with CB only from Day 1 to Day 4; and Group 6, 10 μL solvent control was applied with a micropipette directly to the apical surface of the tissue on Day 0, and then tissues were treated with EGF and CB from Day 1 to Day 4. In Groups 3–6, TEER measurement and tissue histology were performed on Day 4 in the same manner.

**Fig. 4:**
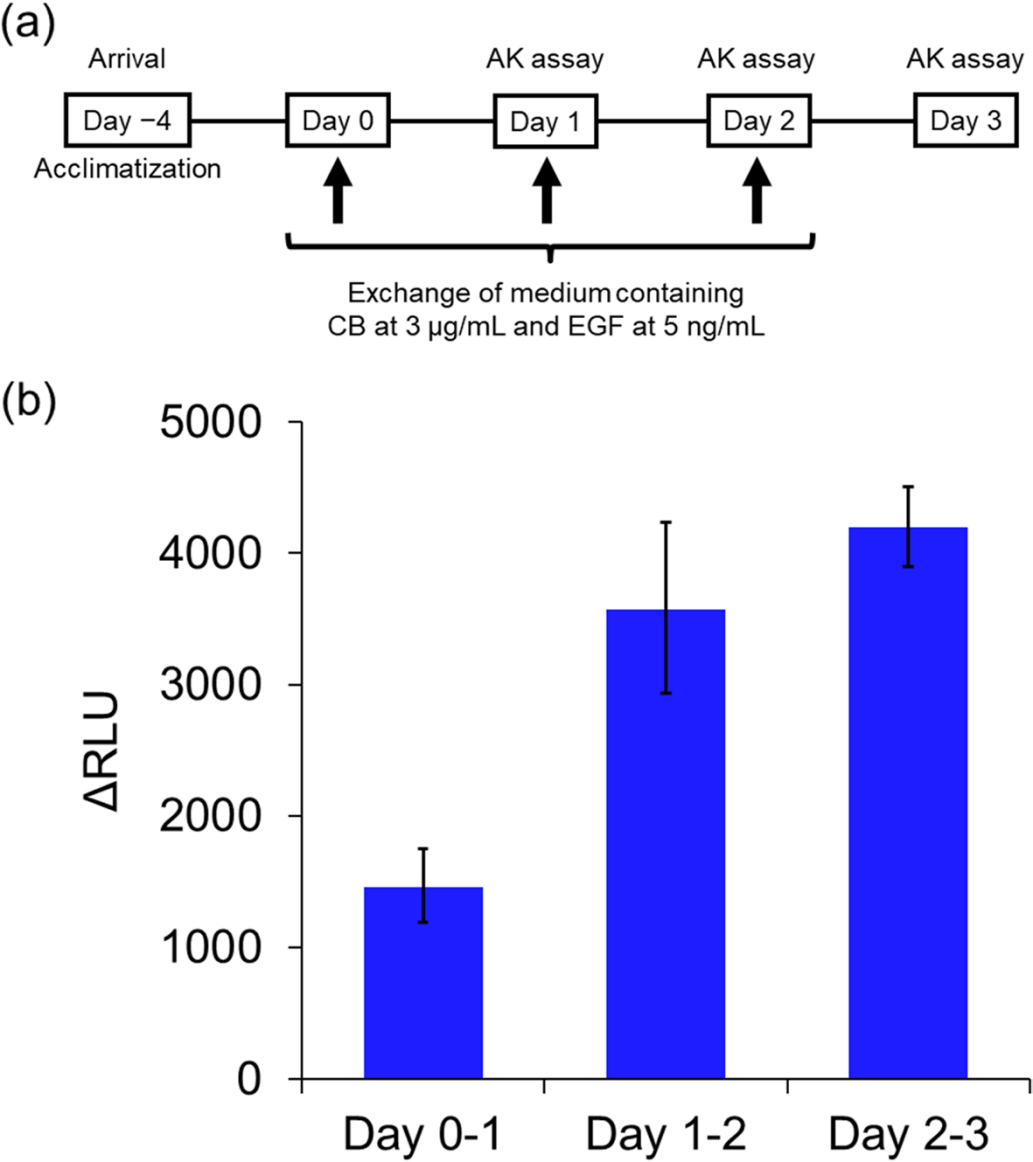
Effect of cytochalasin B incubation and epidermal growth factor stimulation on cytotoxicity. **a** Schematic of incubation with culture medium or with medium containing 3 μg/mL cytochalasin B (CB) and 5 ng/mL epidermal growth factor (EGF) for 72 h in an adenylate kinase (AK) assay. **b** Results of AK assay after incubation with medium containing CB and EGF. Delta relative luminescence units (ΔRLUs) are means ± standard deviations of triplicate inserts

As shown in Fig. 4a, to confirm the effect of CB incubation and EGF stimulation on cytotoxicity, an adenylate kinase (AK) assay was performed. The tissues on the insert were placed in 6-well culture plates and incubated with 1 mL/well of a culture medium in the presence or absence of 3 μg/mL CB and 5 ng/mL EGF on Day 0. Tissues were re-fed twice on Days 1 and 2 with freshly prepared medium using the same procedure as on Day 0. AK assay was performed using the basolateral medium collected on Days 1, 2, and 3.

**Fig. 5:**
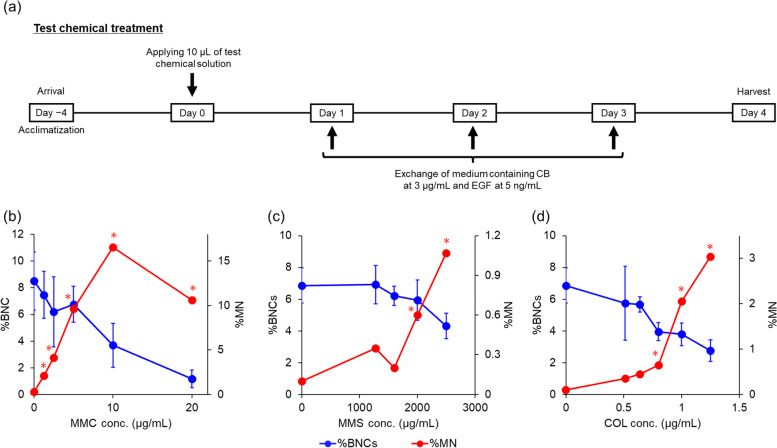
Percentage of binucleated cells and frequency of micronucleated binucleated cells by test chemical treatment. **a** Schematic of test chemical treatment followed by cytochalasin B (CB) incubation and epidermal growth factor (EGF) stimulation. **b**, **c**, and **d** Percentage of binucleated cells (%BNCs) and frequency of micronucleated binucleated cells (%MN) after apical treatment with (b) mitomycin C (MMC), (c) methyl methanesulfonate (MMS), and (d) colchicine (COL) in the EpiAirway™ model. Results are plotted with a blue line indicating %BNCs (left axis) and a red line indicating %MN (right axis). %BNCs are means ± standard deviations of triplicate inserts, while %MN is the combined value of triplicate inserts. *Significantly different from the solvent control group (*p* < 0.05, Fisher’s exact test)

For genotoxicant treatment (Fig. 5a), 10 μL test chemical solution was applied with a micropipette directly to the surface of the tissue on Day 0 without a PBS wash to remove the mucus on the tissue surface. The tissues on the insert were placed in 6-well culture plates and incubated with 1 mL/well of a culture medium containing 3 μg/mL CB and 5 ng/mL EGF on Day 1. Twenty-four hours later, tissues were re-fed twice on Days 2 and 3 with freshly prepared medium containing CB and EGF at the same concentration as on Day 1. Cells were harvested from inserts on Day 4.

### Cell harvesting

For the trypsinization step, each tissue insert was placed in 5 mL DPBS at room temperature for 15 min, then left in 5 mL EDTA (0.1%) at room temperature for 15 min before finally being exposed for 15 min to trypsin–EDTA solution pre-warmed at 37 °C. The detached tissue was transferred to a new well containing 1 mL pre-warmed trypsin–EDTA solution. Then the tissue was rigorously agitated to release additional attached cells. Any cell clumps were disrupted by repeatedly drawing the cell suspension into a pipette and gently expelling the solution. The single-cell suspension (approximately 1.5 mL) was transferred to a 15-mL conical tube containing 1 mL DMEM with 10% FBS to neutralize the trypsin.

### Cell fixation and slide preparation

The cell suspension was centrifuged at 100 × *g* for 5 min and the supernatant was carefully removed. The cell pellet was loosened by gently flicking the bottom of the centrifuge tube, and 1 mL pre-warmed 0.075 M potassium chloride solution was carefully added to the tube. After 3 min, 3 mL freshly prepared cold methanol/acetic acid (3:1 v/v) fixative was slowly added to fix the cells, and then the cell suspension was centrifuged at 100 × *g* for 5 min. After the fixed cell preparation was centrifuged again at 100 × *g* for 5 min, the supernatant was removed. The cell pellet was loosened with the remaining solution and 3 mL cold fresh methanol/acetic acid (99:1 v/v) fixative was added. After the fixed cell preparation was centrifuged again at 100 × *g* for 5 min, the supernatant was carefully removed, leaving 100–200 μL, and the cell pellet was gently resuspended in a smaller volume of the same fixative.

A single drop of the concentrated cell suspension was gently dropped onto a dry slide. After the slides were completely dry, they were immersed in freshly prepared acridine orange solution (final concentration 40 μg/mL in DPBS) for 3 min, immediately rinsed three times with DPBS, and allowed to dry. Stained slides were stored in the dark at 2–8 °C.

### Microscopic observation and parameter calculation

Cells were observed at × 400 magnification by fluorescent microscopy (Olympus BX-51, Tokyo, Japan) equipped with a 400–440 nm excitation filter. In general, 500 cells were scored per tissue to determine the percentage of cells with one, two, and three or more nuclei. The percentage of binucleated cell (%BNCs) were calculated as follows.

$$\%\mathrm{BNC}=\frac{\text{Number of BNCs/total number of cells treated cultures}}{\text{Number of BNCs/total number of cells control cultures}}$$

Calculations of the replication index (RI) and cytokinesis-block proliferation index (CBPI) were performed as previously described by Lorge et al. [25].

RI was determined as:

$$\mathrm{RI}=\frac{\text{(no.BNCs} + 2\times \text{no.multinucleated cells)/total number of cells treated cultures}}{\text{(no.BNCs} + 2 \times \text{no.multinucleated cells)/total number of cells control cultures}}$$

CBPI was determined as:

$$\mathrm{CBPI}=\frac{\text{no.mononucleated cells} + 2\times \text{no.BNCs}+ 3 \times \text{no.multinucleated cells}}{\text{total number of cells}}$$

For the MN analysis, all slides were blindly coded and observed manually. The number of micronucleated BNCs among approximately 2,000 BNCs (approximately 667 cells/slide, triplicate tissues) was counted if possible, and the frequency of micronucleated BNCs (%MN) was calculated. Mitotic cells, multinucleated cells with four or more nuclei, and apoptotic cells were excluded from the MN analysis. The maximum diameter of the MN was defined as half that of the nucleus [26].

### Measurement of transepithelial electrical resistance

TEER was measured to examine tissue integrity using a Millicell® ERS-2 Epithelial Voltohmmeter (Merck Millipore, Billerica, MA, USA) and an ENDOHM-6G chamber (World Precision Instruments, Sarasota, FL, USA). Immediately before TEER measurement, the apical surface of the tissue was rinsed twice with 400 μL TEER buffer. An aliquot of 400 μL TEER buffer was added apically to the tissue insert and left on the apical surface. An aliquot of 500 μL TEER buffer was added to the ENDOHM-6G chamber and the tissue insert was placed within the chamber. The value displayed by the voltohmmeter was multiplied by the insert surface (0.6 cm^2^) to obtain the resistance value of the total area (Ω × cm^2^).

### Histology

Tissues were fixed with 4% paraformaldehyde and embedded in paraffin. Sections prepared from these embedded tissues were subjected to hematoxylin and eosin staining.

### Adenylate kinase assay

AK assay was performed using the ToxiLight™ non-destructive cytotoxicity bioassay kit (Lonza, Switzerland). The basolateral media were collected on Days 1, 2, and 3, diluted with five volumes of AK detection reagent, allowed to incubate for 5 min, and then read in an Infinite 200® PRO instrument (TECAN, Switzerland). The results of the ToxiLight™ non-destructive cytotoxicity bioassay kit were represented as delta relative luminescence units (ΔRLUs). The following equation explains how ΔRLU is calculated: ΔRLU = L_P_ − L_A_, where L_P_ is a unit obtained in the presence of CB and EGF, and L_A_ is a unit obtained in the absence of CB and EGF.

### Statistical analysis

The genotoxicity of each test sample was evaluated as described previously [27]. The Cochran–Armitage trend test was used to examine the dose dependency of %MN. Fisher’s exact test was used to evaluate the significant increase in %MN over the concurrent solvent control. If both the statistical test results were significant at *p* < 0.05, the test sample was considered genotoxic. The data analysis was conducted using JMP (version 16.0.0, SAS Institute Japan, Tokyo, Japan).

TEER data were considered significant at *p* < 0.05 with Welch’s *t*-test against the control.

## Results

### Acquisition of BNCs by CB incubation

Tissues were exposed to various concentrations of CB in the medium and for several incubation periods to determine the optimal concentration and incubation period of CB for the production of BNCs.

We first examined the induction of BNCs under two incubation periods, 48 h and 72 h, at three CB concentrations, 1, 3, and 5 μg/mL (Fig. 1a). As shown in Fig. 1b, a concentration-dependent increase in the percentage of BNCs (%BNCs) was observed for 48 h CB incubation. CB at 5 μg/mL remarkably increased %BNCs (2.4%) compared with no CB incubation (0.1%). However, for 72 h CB incubation, %BNCs peaked at 3 μg/mL (3.7%) then slightly decreased at 5 μg/mL (3.3%).

We further examined the induction of BNCs for 72-h and 120-h incubation periods at two CB concentrations (Fig. 1c). As shown in Fig. 1d, for 72 h CB incubation, 3 μg/mL and 10 μg/mL CB considerably increased %BNCs to 5.1% and 6.3%, respectively, compared with no CB incubation (0.4%). For 120-h incubation, CB incubation at both concentrations increased %BNCs to 5.7% and 6.8%, respectively, compared with no CB incubation (0.3%).

From these results, in which %BNCs almost plateaued at 3 μg/mL CB for 72 h, we chose this concentration and incubation period as the standard conditions for subsequent experiments.

### Cell proliferation stimulated by EGF

Next, to ensure sufficient BNCs for MN analysis, ALI culture medium containing the selected concentration of CB (3 μg/mL) was supplemented with several concentrations of EGF (0, 2.5, 5, and 10 ng/mL) (Fig. 2a).

As shown in Fig. 2b, a concentration-dependent increase in %BNCs by EGF stimulation was observed under CB incubation. %BNCs reached 9.7% at 5 ng/mL EGF, while %BNCs at 5 and 10 ng/mL were almost the same (9.7% and 9.8%, respectively).

From this result, we chose 5 ng/mL as the standard concentration of EGF for subsequent experiments.

### Effect of solvent control treatment, CB incubation, and EGF stimulation on TEER and tissue histology of EpiAirway™

CB, an inhibitor of microfilament assembly, is used to prevent cytoplasmic division after nuclear division [28]. EGF is a ligand of epidermal growth factor receptor and induces bronchial hyperplasia via the mitogen-activated protein/extracellular signal regulated kinase signaling pathway [29–31]. To examine the effects of CB incubation and EGF stimulation on the basic physiological and morphological characteristics of the EpiAirway™ tissue model, TEER and tissue histology were assessed after no treatment (Groups 1 and 2), solvent control treatment (Group 3), EGF stimulation (Group 4), CB incubation (Group 5), and solvent control treatment followed by both CB incubation and EGF stimulation (Group 6) (Fig. 3a).

Statistically significant decreases in TEER values were observed in the tissues of Groups 5 and 6 in which CB incubation was included in the treatment procedures, while any other procedures such as 4-day culture (Group 2), solvent control treatment (Group 3), and EGF stimulation (Group 4) did not affect the TEER values (Fig. 3b). The morphology of EpiAirway™ tissues affected by the various treatments exhibited a thicker tissue layer after EGF stimulation (Group 4) but not with solvent control treatment (Group 3) and CB incubation (Group 5) compared with the no-treatment groups (Groups 1 and 2) (Fig. 3c). This morphological assessment demonstrated that EGF could induce bronchial epithelial cell proliferation. Additionally, we observed several large cyst formations in the treated tissues, including those with CB incubation (Groups 5 and 6), suggesting that CB incubation could lead to unphysiological conditions in the EpiAirway™ tissues.

### Effect of CB incubation and EGF stimulation on cytotoxicity

To confirm the effect of CB incubation and EGF stimulation on cytotoxicity by AK assay, tissues were incubated for 3 days in the presence or absence of 3 μg/mL CB and 5 ng/mL EGF (Fig. 4a). As shown in Fig. 4b, the ΔRLU value was approximately 1500 after the first 24-h incubation period with CB and EGF (Days 0–1). After the second and third incubation periods (Days 1–2 and Days 2–3), the ΔRLU values increased to approximately 3600 and 4200, respectively, demonstrating that CB incubation and EGF stimulation caused tissue damage with each culture day in the EpiAirway™ model.

### MN induction and cytotoxicity by a clastogen and aneugen

**Fig. 6:**
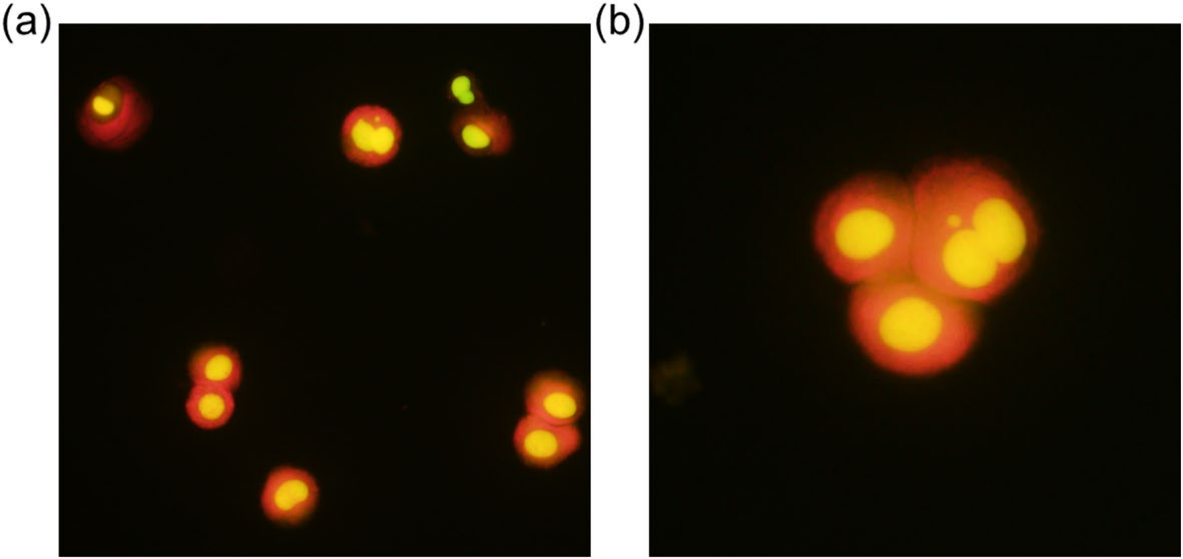
Cell images of an acridine orange-stained slide specimen. **a** Representative example of mononucleated and binucleated cells. **b** Example of a binucleated cell with a micronucleus surrounded by two mononucleated cells

To verify whether a well-studied clastogen and aneugen could be evaluated under the conditions determined in the previous optimization processes, an MN test was performed by treatment with MMC and MMS as the clastogens and COL as the aneugen. To closely mimic the relevant exposure situations that would occur in vivo, EpiAirway™ tissues were treated apically with MMC, MMS, and COL solutions. Twenty-four hours after treatment initiation, tissues were subsequently exposed to 3 μg/mL CB and 5 ng/mL EGF for a further 72 h (Fig. 5a). Acridine orange-stained cell images of mononucleated cells, BNCs, and micronucleated BNCs are depicted in Fig. 6a and b.

First, %BNCs and %MN following MMC treatment were examined (Fig. 5b and Table 1.). A concentration-dependent decrease was found in %BNCs, and was decreased to half at 10 μg/mL (3.7%) compared with the solvent control (8.5%). Furthermore, %BNCs dropped to 1.2% at the highest concentration of 20 μg/mL. As shown in Table 1., the number of BNCs during MN analysis at these two concentrations were 1033 and 217, respectively, which did not achieve the target number of BNCs. %MN showed a statistically significant increase from the lowest concentration of 1.25 μg/mL (2.10%) compared with the solvent control group (0.28%). %MN peaked at 10 μg/mL (16.55%) then decreased at 20 μg/mL (10.60%).

**Table 1:** Effect of well-studied clastogens and aneugen on micronucleus induction and cytotoxicity

Genotoxicants	Concentration (μg/mL)	Number of BNCs observed	Number of BNCs with micronuclei	%MN	%BNCs	%BNCs (relative)	CBPI (relative)	RI (%)
MMC	0.00	1811	5	0.28	8.5	100.0	100.0	100.0
	1.25	2098	44	2.10	7.5	87.7	99.0	87.7
	2.50	2125	88	4.14	6.2	72.7	97.9	72.7
	5.00	1827	176	9.63	6.8	79.4	98.4	79.4
	10.00	1033	171	16.55	3.7	43.4	95.6	43.4
	20.00	217	23	10.60	1.2	13.6	93.2	13.6
MMS	0.00	2001	2	0.10	6.9	100.0	100.0	100.0
	1280.00	2001	7	0.35	7.0	101.7	100.1	101.7
	1600.00	2001	4	0.20	6.2	90.6	99.5	91.9
	2000.00	2001	12	0.60	6.0	87.2	99.2	87.2
	2500.00	1870	20	1.07	4.4	63.8	97.9	66.9
COL	0.00	2001	2	0.10	6.9	100.0	100.0	100.0
	0.51	1975	7	0.35	5.9	85.7	99.1	85.7
	0.64	2001	9	0.45	5.7	82.7	98.9	82.7
	0.80	2001	13	0.65	4.0	57.9	97.3	57.9
	1.00	1555	32	2.06	3.8	55.5	97.5	60.8
	1.25	593	18	3.04	2.8	40.1	96.6	46.9

We then investigated the effect of MMS treatment on %BNCs and %MN. A concentration-dependent decrease was found in %BNCs. %BNCs was 4.4% at the highest concentration of 2,500 μg/mL, which was slightly lower than the solvent control (6.9%). As for %MN, a concentration-dependent increase was observed. At the two highest concentrations of 2,000 and 2,500 μg/mL, %MN values were 0.60% and 1.07%, respectively, which were significantly higher than those of the solvent control group (0.10%).

The effect of COL treatment on %BNCs and %MN was then examined. A concentration-dependent decrease was found in %BNCs up to the highest concentration of 1.25 μg/mL (2.8%) compared with the solvent control group (6.9%). At the two highest concentrations, the number of BNCs were 1555 and 593, as shown in Table 1., which again did not fulfill the target number of BNCs to be observed for MN analysis. COL also induced statistically significant increases in %MN at the three highest concentrations (0.65% at 0.80 μg/mL, 2.06% at 1.00 μg/mL, and 3.04% at 1.25 μg/mL).

For the measurement of cytotoxicity, two methods are recommended for the in vitro MN test when using CB as per OECD Test Guideline 487 [19]. One is based on CBPI and the other is the RI. We then calculated not only %BNCs but also CBPI and RI for the cytotoxicity assessment in the EpiAirway™ model, as shown in Table 1.

As for %BNCs and RI, a concentration-dependent decrease was clearly observed following treatment with each genotoxicant. The relative ranges of %BNCs and RI were both 13.6–101.7%. In contrast, that range of CBPI was quite narrow, whereby CBPI values ranged from 93.2 to 100.1%.

## Discussion

In this study, we used the EpiAirway™ organotypic human airway tissue model to demonstrate that an MN test can be used to detect the potential of a well-studied clastogen and aneugen to induce MN under optimized CB incubation and EGF stimulation conditions. We also found that chemical treatment should be undertaken prior to CB incubation to prevent damage to the organotypic human airway model. These findings suggest that highly differentiated tissue models of the human airway, which typically have less active cell proliferation, can be applied to evaluate the MN-inducing potential of chemicals under optimal conditions in which EGF stimulates cell division and CB identifies the BNCs immediately after chemical treatment of the apical surface. To our knowledge, this is the first study to demonstrate that the assessment of the clastogenic and aneugenic potential of chemicals can be measured in an organotypic human airway model, which expands the genotoxicity endpoints that can be assessed through inhalation exposures.

We first optimized the CB concentration and incubation period to obtain as many BNCs as possible in our organotypic human airway model. Figure 1b and d show that a plateau in %BNCs was reached at approximately 3 μg/mL CB at 72-h incubation with medium exchanged every 24 h, based on the present optimization process using a range of 1–10 μg/mL CB and three incubation periods of 48, 72, and 120 h. This suggests that a higher concentration of CB over a longer incubation period may not linearly increase the %BNCs in EpiAirway™, consistent with previous studies in human peripheral blood lymphocytes [32] and Huh6, a human-derived liver cell line [33], which found that a higher concentration of stimulant over a longer incubation period did not produce a linear increase in cell proliferation.

To increase the low spontaneous rate of cell division in organotypic human airway models, we implemented EGF stimulation during CB incubation and observed an increase in %BNCs of approximately 2.5-fold compared with CB alone, as indicated in Fig. 2b. However, EGF stimulation was not as effective as the in vitro MN test [21–23] using human HepaRG cell lines. Additionally, Lee et al. reported that EGF could only stimulate cell proliferation at the basal cell layer, and not in other cell types such as ciliated and goblet cells [24], indicating that MN induction in the EpiAirway™ model with EGF stimulation has an effect limited to the basal cells. Despite these points, we regarded this procedure as useful for the shorter observation time needed to score %BNCs and %MN on a slide. Therefore, we chose the simultaneous conditions of CB incubation and EGF stimulation in our organotypic human airway model.

In this study, we also examined the effect of CB incubation and EGF stimulation on the physiological and structural characteristics of the EpiAirway™ model. Our results revealed that these procedures reduced barrier integrity, caused cyst formation, and loosened tight junctions. Our observations regarding the effects of CB incubation on reduced barrier integrity and loosened tight junctions are consistent with previous research showing that CB treatment in a corneal model disrupts actin microfilaments, which are a major component of the cytoskeletal system and are present near tight junctions, leading to reduced electrical resistance [34]. Other research has also found that CB treatment increases tight junction permeability in an intestinal epithelial model using Caco-2 cell lines [35]. These findings suggests that cyst formation in the multi-layered tissue structure of organotypic human airway models may be related to loosened tight junctions. In contrast, it has been reported that there are no morphological changes in the organotypic human reconstructed skin tissue of EpiDerm™ after CB incubation [36]. The varying effects of CB incubation on tissue morphology between EpiDerm™ and EpiAirway™ may be due to the different rates of cell proliferation. In the EpiDerm™ model with a higher rate of cell proliferation (in which approximately 50% of the cells are undergoing cell division), the vulnerability of cell-to-cell adhesion due to CB incubation may be exacerbated by the presence of the generated daughter cells. However, in our organotypic human airway model with only approximately 4% of BNCs, this reinforcement may not occur, resulting in the formation of cysts.

Furthermore, the AK assay was performed to examine the potential impact of CB incubation and EGF stimulation on cytotoxicity. As shown in Fig. 4b, an increase in ΔRLUs resulting from AK release was observed during CB incubation with EGF stimulation. This cytotoxicity is believed to be a result of CB incubation alone, as observed in Group 5, which caused cyst formation and disrupted tight junctions, as illustrated in Fig. 3b and c. On the basis of the cytotoxicity observed in Fig. 4 and TEER reduction and cyst formation depicted in Fig. 3, it was suggested that CB incubation and EGF stimulation should not be performed simultaneously during chemical treatment to maintain the normal physiological and structural features of the EpiAirway™ tissue, ensuring accurate chemical absorption and metabolism.

On the basis of the optimal conditions for CB incubation and EGF stimulation in the EpiAirway™ model, we found that MMC, MMS, and COL, which are representative chemicals with different mechanisms of genotoxic action, induced micronuclei in a concentration-dependent and statistically significant manner under these conditions. There is limited genotoxicity testing data available for inhalation exposure in vivo owing to the cost and specialized equipment required for this exposure route. Therefore, the current organotypic human airway model has potential in the genotoxic assessment of chemical exposure through inhalation.

It is critical to establish cytotoxicity criteria to exclude confounding factors in the assessment of genotoxicity of chemicals. In this study, we determined %BNCs, CBPI, and RI based on the frequency of mononucleated, binucleated, and multinucleated cells observed on slides. As shown in Table 1., known genotoxicants caused concentration-dependent decreases in any cytotoxicity index. However, the decrease in CBPI was less significant compared with other indices. This may be due to the fact that CBPI takes into account the number of mononucleated cells, and that small changes in binucleated and multinucleated cells do not significantly affect its calculation in the human airway tissue model where cell division is low. Therefore, in addition to cell division-based cytotoxicity indices such as %BNC and RI, other methods of assessing cytotoxicity, such as TEER measurements and histological analysis (illustrated in Fig. 3), as well as AK assay (shown in Fig. 4), may be useful as cytotoxicity criteria in our tissue model.

Additionally, the low rate of cell proliferation exacerbates the already time-consuming nature of manual MN counting. To address this issue, high throughput observation approaches using imaging flow cytometry and deep learning image classification have recently been reported in cytokinesis-block micronucleus (CBMN) tests in human lymphoblastoid TK6 cells [37] and in reconstructed human skin EpiDerm™ cells [38]. Another approach involves a CBMN test combined with a convolutional neural network to create software for rapid standard automated detection of micronuclei in Giemsa-stained binucleated lymphocyte images [39]. To address the aforementioned issue and improve statistical power and reproducibility, automated detection of MN has become a critical topic.

To validate the future use of this model, validation experiments will be conducted to determine the effectiveness of multiple exposures, the ability to detect genotoxic substances requiring metabolic activation, the results with apoptosis-inducing chemicals, and the feasibility of exposure to gases and aerosols. First, a single exposure prior to CB incubation and EGF stimulation was sufficient to detect the MN-inducing potential of the three genotoxicants tested in this study. However, it is possible that other genotoxic chemicals may require multiple exposures to reveal their genotoxic effects, as previously demonstrated by Wang et al. in their organotypic human airway model, in which the genotoxic potential of ethyl methanesulfonate was evaluated through repeat exposure over a 28-day period [17]. Second, chemicals that require metabolic activation to exert their genotoxic effects should also be evaluated because previous research has shown that several critical xenobiotic-metabolizing enzymes, including cytochrome P450 (CYP) 1A1, CYP1B1, and CYP2A6, are present in the EpiAirway™ [40, 41] and MucilAir™ [42] models. Third, organotypic human airway models should be used to evaluate chemicals that cause cytotoxicity such as apoptosis, but do not produce the genotoxicity, to demonstrate the validity of this model. Finally, although the test chemical was dissolved in an aqueous solvent in this study, exposure to gaseous or aerosol conditions would be more representative of inhalation exposure in humans.

The present study represents the initial step in the development of a MN test system using an organotypic human airway model cultured under ALI conditions. It is hoped that future investigations utilize this model to assess the genotoxic potential of chemical exposure by inhalation in humans.

## Conclusions

The present study found that EGF stimulation with CB incubation in the EpiAirway™ model resulted in an increase in the percentage of BNCs. These experimental conditions also allowed for the detection of dose-dependent induction of MN by clastogens and an aneugen in this organotypic human airway model cultured under ALI conditions. Our results demonstrate the possibility of using this MN test system to assess the genotoxic potential of chemical exposure by inhalation in humans.

## Data Availability

All data generated or analyzed in this study are included in the published article. All materials used in this study are described in the article.

## Abbreviations

3D: Three-dimensional
AK: Adenylate kinase
ALI: Air–liquid interface
BNCs: Binucleated cells
%BNCs: Percentage of binucleated cells
CB: Cytochalasin B
CBPI: Cytokinesis-block proliferation index
COL: Colchicine
CYP: Cytochrome P450
DMEM: Dulbecco’s modified Eagle medium
DPBS: Dulbecco’s phosphate buffer saline
EDTA: Ethylenediaminetetraacetic acid
EGF: Epidermal growth factor
FBS: Fetal bovine serum
MMC: Mitomycin C
MMS: Methyl methanesulfonate
MN: Micronucleus
%MN: Frequency of micronucleated binucleated cells
OECD: Organization for Economic Co-operation and Development
RI: Replication index
ΔRLU: Delta relative luminescence unit
SC: Solvent control
TEER: Transepithelial electrical resistance
WG: Working group
